# Alarm communication networks as a driver of community structure in African savannah herbivores

**DOI:** 10.1111/ele.13432

**Published:** 2019-11-27

**Authors:** Kristine Meise, Daniel W. Franks, Jakob Bro‐Jørgensen

**Affiliations:** ^1^ Mammalian Behaviour and Evolution Group Department of Evolution, Ecology and Behaviour Institute of Integrative Biology University of Liverpool Neston CH64 7TE UK; ^2^ Department of Biology University of York York YO10 5DD UK; ^3^ Department of Computer Science University of York York YO10 5GH UK

**Keywords:** Agent‐based modelling, antipredator strategies, interspecific information transfer, mixed‐species groups, resource competition, social affinity, social information use, social network analysis, spatial ecology, ungulates

## Abstract

Social information networks have the potential to shape the spatial structure of ecological communities by promoting the formation of mixed‐species groups. However, what actually drives social affinity between species in the wild will depend on the characteristics of the species available to group. Here we first present an agent‐based model that predicts trait‐related survival benefits from mixed‐species group formation in a multi‐species community and we then test the model predictions in a community‐wide field study of African savannah herbivores using multi‐layered network analysis. We reveal benefits from information transfer about predators as a key determinant of mixed‐species group formation, and that dilution benefits alone are not enough to explain patterns in interspecific sociality. The findings highlight the limitations of classical ecological approaches focusing only on direct trophic interactions when analysing community structure and suggest that declines in species occupying central social network positions, such as key informants, can have significant repercussions throughout communities.

## Graphical abstract

Ecological theory has traditionally viewed community structure as the outcome of direct trophic interactions. We here combine agent‐based modelling of mixed‐species group formation with a field study of African savannah herbivores to show that also social interactions between species, such as social information use, can drive the spatial structure of communities. The study highlights that declines in species occupying central social network positions can have community‐wide repercussions.

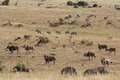

## Introduction

Ecological theory has traditionally viewed community structure and function as the outcome of direct trophic interactions such as predation, herbivory, and interspecific resource competition (Morin 2011). Over the past decade, however, attention has increasingly turned to the fact that also information exchange between species can provide critical benefits that may substantially affect social attraction and fitness (Goodale *et al.* 2010, 2017; Parejo & Avilés 2016; Gil *et al.* 2017, 2018; Sridhar & Guttal 2018). It is only recently that a general theoretical framework has been developed which integrates the benefits of social information use with costs and benefits from direct trophic interactions, and it predicts that social information use in general will promote the formation of mixed‐species groups (MSGs) (Gil *et al.* 2017). Yet, by using a single generalised phenotype to represent the option of grouping with heterospecifics, the framework does not capture the fact that animals in the wild typically have the choice between grouping with a wide range of heterospecifics that often vary dramatically in their species characteristics. This variation can be expected to have significant consequences for heterospecific attraction and whether MSGs form, with both the maximum benefits obtained from the most attractive heterospecific, and the proportion of heterospecifics that are more attractive than conspecifics, potentially having an effect. Empirically, although its fitness benefits have only been scarcely studied, MSG formation has been linked to increased survival, but again the mechanisms involved are often unclear (Dolby & Grubb 1998; Jullien & Clobert 2000; Goodale *et al.* 2017). Community‐wide studies that encompass the range of options for MSG formation available in natural systems are therefore essential to further our understanding of the role of social information use relative to other predation‐ and resource‐related drivers in shaping the social attraction between species. Here we fill this gap by combining a theoretical and an empirical approach to analyse patterns in social attraction between species at the community level.

The principles behind grouping behaviour have so far overwhelmingly been studied in single‐species systems (Krause & Ruxton 2003; Székely *et al.* 2010), that differ from multi‐species systems in which payoffs from sociality depend fundamentally on variation between species in traits that affect costs and benefits from predation and resource acquisition (Goodale *et al.* 2017). Similar to single‐species groups, antipredator benefits of MSG formation can be expected to depend on how much the probability of detecting an approaching predator is increased by social information use, i.e. detection benefits (‘many eyes effect’; Pulliam 1973), and how much the probability of the focal individual being targeted during a predator attack is reduced, i.e. dilution benefits (Hamilton 1971). However, in MSGs, detection benefits are likely to further depend on (1) the ability of heterospecifics to detect predators, (2) the probability that they alarm call in response to predators detected, and (3) the relevance of the heterospecific alarm call to the receiver, which in turn depends on the overlap in predators between the alarm caller and the receiver (Magrath *et al.* 2015). Dilution benefits, on the other hand, are likely to depend on the vulnerability of heterospecifics to any shared predators. Thus, whereas dilution benefits for single‐species systems are generally considered to be a simple function of group size (Treisman 1975; Krause & Ruxton 2003), for MSGs, the number of heterospecifics must be weighted by their predator vulnerability relative to that of conspecifics, which may be either higher or lower. Because species providing high dilution benefits may provide only modest detection benefits (and *vice versa*), and species providing high antipredator benefits may simultaneously incur high resource competition costs, trade‐offs between these costs and benefits will affect patterns in social affinity between species (Goodale *et al.* 2010; Gil *et al.* 2017; Sridhar & Guttal. 2018). Understanding how different species characteristics interact to determine the payoffs from forming MSGs is therefore central to identifying the principles underlying the spatial structure of ecological communities.

**Figure 1:**
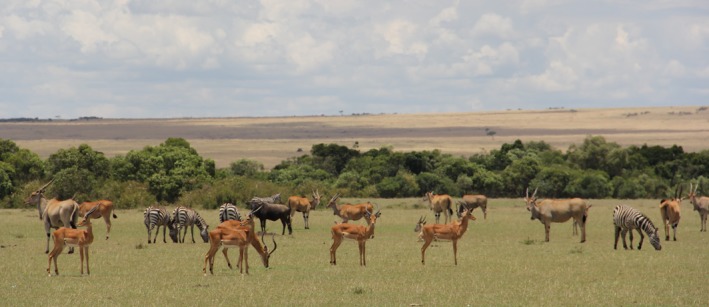
Mixed‐species group of zebras (*Equus quagga*), eland (*Tragelaphus oryx*), wildebeest (*Connochaetes taurinus*) and impala (*Aepyceros melampus*) in the Masai Mara study system (Masai Mara National Reserve, Kenya; photo: Jakob Bro‐Jørgensen).

Savannah herbivores present an example of a community dominated by MSGs where heterospecifics have the potential to both provide protection against predators and act as competitors for food (Sinclair 1985; Stensland *et al.* 2003). Previous studies have found interspecific grouping patterns in this system to be non‐random, however, the principles behind the assortative processes remain obscure (Kiffner *et al.* 2014). In a community‐wide study of African savannah herbivores, we here examine the species characteristics that determine social affinities between species (Fig. 1). We first develop an agent‐based model (ABM) to predict the survival probability of an imaginary focal individual when joining groups of either conspecifics or various heterospecifics that differ in their characteristics. In a large‐scale ecological field study on the 12 most common herbivore species of the open plain habitat in the Masai Mara ecosystem (Ogutu *et al.* 2011; Meise *et al.* 2018), we then use multi‐layered network analysis to test the predictions generated by the ABM regarding the roles of traits related to detection benefits, dilution benefits and resource competition costs in determining social affinity.

## Methods

### Agent‐based model

**Figure 2:**
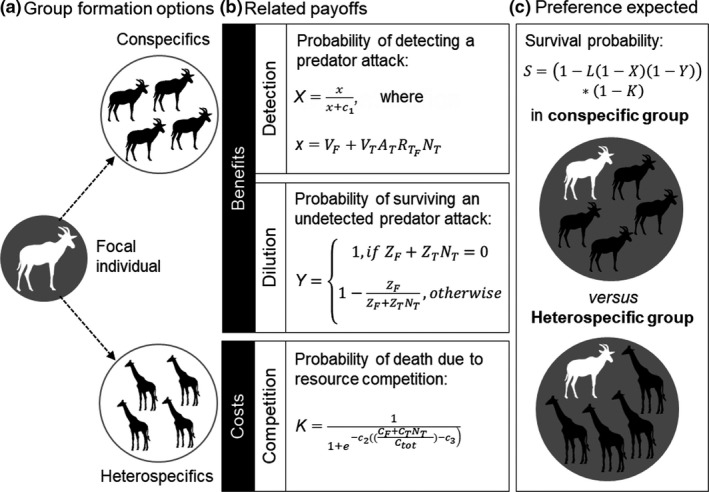
Agent‐based model of payoffs from joining heterospecifics compared to conspecifics. (a) The model allocates a focal individual *F* to a group of target individuals *T*, which are either conspecifics or heterospecifics. Conspecifics share the traits of the focal individual, but heterospecifics can have different values for vigilance *V*, probability of alarm calling *A*, predator vulnerability *Z*, resource consumption *C*, and the relevance of their alarm call to the focal individual *R*. (b) Detection benefits *X*, dilution benefits *Y* and resource competition costs *K* emerge from these species‐specific traits and the number of group members *N*; *c_1_*, *c_2_*, *c_3_*, and *C_tot_* are constants where the latter denotes the total amount of resources available. (c) The survival probability *S* of the focal individual in a given group is then calculated based on the anti‐predator benefits and the resource competition costs as well as the probability of the group being targeted by a predator *L*.

The ABM explicitly represents a single individual of a focal species *F* as an agent that can group with individuals of one of several potential target species *T* and is preyed upon by several predator species *P* (see Table S1 for a reference list of symbols). The target group can consist of either conspecifics or one of several heterospecific species, where conspecifics share the characteristics of the focal individual and heterospecifics differ in one or more of the characteristics (Fig. 2). The model allocates individuals to either a conspecific or a heterospecific group as follows:

1. Selects the focal species – the species of the individual for whom survival probability is calculated.
2. Chooses the target species – the species with which our focal species will be grouping; this can be the conspecific or a heterospecific species.
3. Selects a group size at random from the target species’ group‐size distribution and adds that number of target individuals to the group.

#### Probability of death due to predation

The predator‐specific predation pressure exerted on the prey community is assumed to depend on the predator’s proportion of the total biomass of all prey consumed *Q_P_*, which in turn depends on the abundance of the predator and its meat consumption rate *per capita.* This measure is used to weight the impact of encounters with different predators on the prey community in general. The probability of a predator selecting a group as a target upon encounter is assumed to depend on the predator’s preference for the most preferred prey in the group:

$$L_{P}=\max\left(Z_{P_{F}},Z_{P_{T}}\right),$$

where Z*_P_* is the vulnerability of prey to the predator.

In modelling detection benefits, the probability of the focal individual escaping an attacking predator is assumed to depend on the probability of the focal individual detecting the predator, which is expressed as a function of the focal individual’s own vigilance and information received from group members, the latter in turn depending on vigilance of the group members, the probability that they alarm call upon detecting the predator, the relevance of the alarm call to the focal individual and the group size:

$$x_{P_{F}}=V_{F}+V_{T}A_{P_{T}}R_{T_{F}}N_{T},$$

where *V* is vigilance, $A_{P_{T}}$ is the probability of the target species alarm calling to predator *P*, $R_{T_{F}}$ is the relevance of the alarm call of the target species to the focal individual, and *N* is the group size. The following transformation gives a value between 0 and 1:

$$X_{P_{F}}=\frac{x_{P_{F}}}{x_{P_{F}}+c_{1}},$$

where *c_1_* is a constant determining the rate of approaching the asymptote.

To model dilution benefits, the probability of the focal individual not being killed when an undetected predator attacks is expressed as a function of its own predator vulnerability relative to that of the other group members and the group size:

$$Y_{P_{F}}=\left\{\begin{array}{c} 1,\text{if}\;Z_{P_{F}}+Z_{P_{T}}N_{T}=0\\ 1-\frac{Z_{P_{F}}}{Z_{P_{F}}+Z_{P_{T}}N_{T}},\text{otherwise} \end{array}\right.$$

The overall probability of death due to predation depends on the above as follows:

$$D_{F}=1-\prod_{p=1}^{p}\left(1-Q_{P}L_{P}\left(1-X_{P_{F}}\right)\left(1-Y_{P_{F}}\right)\right),$$

where *p* is the number of predator species.

#### Probability of death due to resource competition

The resource competition cost for the focal individual in a given group is assumed to depend on the proportion of resources at the patch which is consumed by the group as follows:

$$k_{F}=\frac{C_{F}+N_{T}C_{T}}{C_{\mathrm{tot}}},$$

where *C* is the individual resource consumption and *C_tot_* the total amount of resources available. This proportion is transformed to a value between 0 and 1 by a logistic function:

$$K_{F}=\frac{1}{1+e^{-c_{2}\left(k_{F}-c_{3}\right)}},$$

where *c_2_* and *c_3_* are constants.

#### Survival probability

The overall survival probability of the focal individual in a given group then depends on the probability of death due to predation and resource competition as follows:

$$S_{F}=\left(1-D_{F}\right)\left(1-K_{F}\right)$$

#### Parameterisation and analysis of model outputs

We systematically varied parameters for the focal and target species to understand how the costs and benefits of mixed‐species grouping depend on species‐specific characteristics (Table S2). We initially modelled a total of 421 875 survival probabilities resulting from different combinations of characteristics for the focal and the target species. For simplicity, we here present only results relating to focal species with the low, intermediate and high trait values (0, 0.5, 1), with more detailed variation in trait values for target species (0, 0.25, 0.5, 0.75, 1); group size of con‐ and heterospecifics was assigned from the same distribution, which fell within the natural range observed in the study system, as specified in Table S2. This resulted in 1875 trait combinations of the target species for each of 162 trait combinations of focal species. To facilitate the interpretation of the model outputs, we considered only one predator, hence *Q* = 1. *c*_1_ was arbitrarily set to 0.8 to obtain an intermediate approach rate to the asymptote. *c*_2_ was set to 10 to obtain a smooth logistic function, rather than a linear or stepwise function. *c*_3_ was set to 0.5 to ensure the inflection point of the logistic function was centred at 0.5. For each focal species, we calculated two statistics which could promote MSG formation: (1) the proportion of the heterospecific species with whom grouping resulted in higher survival probability than experienced in the typical conspecific group, and (2) the change in survival probability experienced by the focal individual when grouping with the heterospecific species that yielded the highest survival probability compared to when grouping with conspecifics.

### Field study

#### Study system

The field study was conducted in the Masai Mara National Reserve, southern Kenya (1°30’S, 35°10’E), which is part of the larger Serengeti‐Mara ecosystem. The study species were the 12 most common herbivore species of the open plain habitat: Thomson’s gazelle (*Gazella thomsonii*), Grant’s gazelle (*Gazella granti*), impala (*Aepyceros melampus*), common warthog (*Phacochoerus aethiopicus*), ostrich (*Struthio camelus*), topi (*Damaliscus lunatus*), hartebeest (*Alcelaphus buselaphus*), blue wildebeest (*Connochaetes taurinus*), plains zebra (*Equus quagga*), African buffalo (*Syncerus caffer*), common eland (*Tragelaphus oryx*) and giraffe (*Giraffa camelopardalis*) (Ogutu *et al.* 2011). Of these, wildebeest and zebra perform a large‐scale annual migration between the Masai Mara National Reserve in the north and the Serengeti National Park in Tanzania to the south (Sinclair *et al.* 2008). The five main predators of the study species are lion (*Panthera leo*), leopard (*Panthera pardus*), cheetah (A*cinonyx jubatus*), spotted hyena (*Crocuta crocuta*) and black‐backed jackal (*Canis mesomelas*) (Sinclair *et al.* 2003). The hunting success of these predators depends on the delaying detection of their predatory intent, which reduces the chance of their prey escaping, with the hunting strategies of the cats relying on ambush and stalking, the hyena being a cursorial predator, and the jackal using various strategies, notably stalk‐and‐pounce (Estes 2011). The main field study was conducted between September 2015 and October 2016, preceded by alarm call acquisition for the playback experiments during earlier fieldwork in the study area.

### Quantification of species characteristics

Data are available from the NERC Environmental Information Data Centre (EIDC; Bro‐Jørgensen *et al.* 2019a) and the Dryad Digital Repository: https://doi.org/10.5061/dryad.mb7dd20.

**Vigilance (*V*)** was determined as the proportion of time spent vigilant. For this purpose, we video‐recorded monospecific groups during otherwise uninterrupted foraging bouts. For 574 individuals in a total of 109 groups, we recorded the duration of instances when the head was lifted above the shoulder level over a 5‐min period. To obtain comparable values for the different species, we focussed the data collection on groups of similar size of approximately 4–10 individuals (group size: 7.6 ± 0.32, mean ± SE). We calculated the mean proportion of time that individuals spent vigilant as no significant effect of the limited variation in group size was found in a general linear model with species included as an independent variable (Table S3).

**Probability of alarm calling (*A*)** was determined from predator simulation experiments (*N* = 651) in which we presented the study species to life‐sized photostats of their five main predators in the lateral view (see Meise *et al.* 2018 for details). We recorded the occurrence of alarm calls during the first 5 min following detection of the dummy. We constructed a generalised linear model with binomial distribution and logit link function to model predator‐specific alarm call probabilities for each study species, using the occurrence of alarm calls as the response variable and including species identity as a fixed factor, while also controlling for the distance between the model and the focal individual as a covariate and the presence of young in the group as a fixed factor (Table S3). The predicted values were summed for each study species to provide an index.

**Relevance of alarm calls (*R*)** was quantified from playback experiments (*N* = 2434 playbacks) (see Meise *et al.* 2018 for details on playback design). In brief, alarm calls of the study species were played back to foraging individuals in a round robin design. We measured presence/absence of a response (defined by the head being raised within 10 s after the playback), response latency, response duration and head‐lifting speed, using the Behavioural Observation Research Interface (BORIS) Software (Friard & Gamba 2016). These four variables were analysed in a principal component analysis and the first principal component, which explained 94% of the variation, was then modelled as the response variable in a general linear model with species ID as a fixed factor and grass height, wind speed and speaker distance as covariates. The predicted values were used to quantify the response strength in an interspecific communication network (Table S4).

**Body mass (*M*)** was measured as the mean between the mass of adult males and females reported in the literature (in kg; mammals: Estes 2011; ostrich: Deeming *et al.* 1996; Table S3).

**Group size (*N*)** was determined from 66 species counts conducted at regular intervals over a full year on three study plains covering an area of 57 km^2^. From a Landcruiser 4WD, the location and species identity of all individuals were recorded using a GARMIN GPS receiver (Oregon 600) and a laser rangefinder (Bushnell Scout DX 1000 ARC). Groups were defined by inter‐individual distance < 100 m (Meise *et al.* 2018). Species‐specific group size was determined as the mean of all monospecific groups recorded (Table S3).

**Diet overlap (*O*)** between species was calculated based on the Pianka index of niche overlap (Pianka 1973):

$$\begin{array}{c} O_{\mathrm{FT}}=\frac{\sum_{r=1}U_{r_{F}}U_{r_{T}}}{\sqrt{\sum_{r=1}U_{r_{F}}^{2}\sum_{r=1}U_{r_{T}}^{2}}}, \end{array}$$

where *u_r_* is the proportion of the resource *r* in the diet, where *r* was graze or browse (Bro‐Jørgensen 2013; Table S3).

**Habitat overlap (*H*)** between any two species was estimated by the numerical difference in normalised difference vegetation index (NDVI) of their sites of occurrence at 500 × 500 m resolution (Pettorelli 2013; Didan 2015). To obtain the most representative NDVI value for each species over the course of the study, we calculated the mean across all individuals sighted during the species counts, which were conducted at 16‐day intervals to match the temporal resolution of MODIS‐NDVI data (Table S3).

**Similarity in movement pattern (migratory/resident) (*J*)** was coded as ‘0’ when one species was migratory and the other resident and ‘1’ when both species were either migratory or residents.

**Phylogenetic relatedness (*E*)** was controlled for based on the phylogeny in Fernández & Vrba (2005) for the ruminants, Gatesy *et al. *(2013) for the other mammals, and Benton (1990) for the ostrich (Table S5).

**Predator‐specific predation pressure (*Q_P_*)** on the prey community was estimated by the predator’s proportion of the total meat consumption, which was calculated from the local abundance of the five main predators (Broekhuis 2016) and their average daily meat consumption *per capita* (Mills *et al.* 1993; except for the jackal, for which the value was extrapolated).

**Social affinity (*W*)** within species pairs was determined by using the data from the species counts to calculate the social affinity index according to Meise *et al. *(2019). This index takes into account the number of individuals of each species in each group as well as the relative abundance of all species in the population:

$$W_{F_{T}}=\left(\sum_{t=1}^{g}\frac{N_{i_{T}}}{\left(N_{i}-1\right)}*N_{i_{F}}\right)*\frac{1}{\sum N_{F}}*\frac{\sum N-1}{\sum N_{T}},$$

where *g* is the number of groups, and *N_i_* is the number of individuals in the group *i* (note the subtraction of 1 is to exclude the focal individual). Following Godde *et al. *(2013), the affinity index was controlled for the gregariousness of each species:

$$w_{F_{T}}=W_{F_{T}}\frac{\sum W}{\sum W_{F}\sum W_{T}},$$

where ∑*W_F_* and ∑*W_T_* are the sums of the social affinity indices of the focal and target species, respectively (i.e. a measure of their gregariousness), and ∑*W* is the sum of the social affinity indices of all species. The affinity indices were used to create an undirected social affinity network for the study community (Table S6). Direction of the relationships within this network was weighted by assuming that the likelihood of each species in a dyad driving the formation of the MSGs could be estimated from their relative payoffs predicted by the ABM model when parameterised with data from the study system (Table S2). For each dyad in the social affinity network, we thus calculated the expected survival probability of an individual of each species when in a group of the other species relative to when solitary; we then multiplied the undirected affinity index by each species’ proportion of the sum of the dyad’s payoffs to obtain a directed social affinity network (Table S7). In these calculations, we explicitly accounted for the five main predators of the study species by using predator‐specific values for alarm call probability *A* and predator vulnerability *Z* of each study species, the latter based on prey preferences reported in the literature (Hayward & Kerley 2005; Hayward 2006; Hayward *et al.* 2006a, 2006b, 2017; Table S3). The probability distribution for monospecific group sizes was calculated from a generalised linear model assuming a Poisson distribution with the observed frequency as the response variable and the log‐transformed group size as a covariate and species ID as a fixed factor. Resource competition was quantified by multiplying the diet overlap between the focal and target species by the food intake of the target species, calculated from Kleiber’s law (Kleiber 1975; Demment & van Soest 1985):

$$k_{F_{T}}=O_{\mathrm{FT}}M_{T}^{0.71}$$

For further details on parameterisation, see Tables S2 and S3.

### Statistical analysis of affinity drivers

Due to the non‐independence of the dyadic data (Croft *et al.* 2011), we used a multiple regression quadratic assignment procedure (MRQAP) with double‐semi partialing (DSP) to establish the statistical significance of the relationship between species‐specific characteristics and mutual affinity, with DSP being robust against multicollinearity (Krackhardt 1988; Dekker *et al.* .2007; Farine 2013). In a multi‐layered social network analysis, we modelled the directed affinity network as a function of vigilance, alarm call probability and relevance, log‐transformed body mass, log‐transformed group size and diet overlap, with control for habitat overlap, similarity in movement pattern, and phylogenetic relatedness as follows:

$$w_{F_{T}}∼V_{F}*V_{T}+A_{P_{F}}*A_{P_{T}}+R_{F_{T}}+M_{F}*M_{T}+N_{F}*N_{T}+O_{\mathrm{FT}}+H_{\mathrm{FT}}+J_{F_{T}}+E_{F_{T}}$$

All variables were standardised to range between 0 and 1. In a multiple‐regression approach, the final model was obtained by removing all fixed effects with *P *> 0.1 that resulted in ΔAIC > 2, starting with all interactions. The model fit was checked by visually confirming (1) homoscedasticity in a plot of fitted versus residual values and (2) the normal distribution of the residual frequency distribution in a QQ plot. We also ran the analysis with the undirected network as the response variable to assess if the results were affected by our approach of directing the network by using empirical data to inform the ABM (see above); the only qualitative difference was that the effect of phylogeny became a tendency rather than statistically significant, confirming the general reliability of the approach (cfr. Table 1 and Table S8).

**Table 1:** Species characteristics as predictors of social affinity for heterospecifics within the African savannah herbivore community (Masai Mara NR)^a^

Trait	Coefficient	*P*‐value	Related costs/benefits
Vigilance, focal sp.	**−0.237**	0.014*	*Detection benefits*
Vigilance, target sp.	**−**0.121	0.131	*Detection benefits*
Vigilance, focal sp. × vigilance, target sp.	0.303	0.069	*Detection benefits*
Responsiveness to alarm call of target sp.	**0.166**	0.026*	*Detection benefits*
Alarm call probability, focal sp.	**−**0.113	0.071	*Detection benefits*
Alarm call probability, target sp.	**−**0.079	0.164	*Detection benefits*
Body size, focal sp.	**−0.155**	0.039*	*Dilution benefits; resource competition costs*
Body size, target sp.	**−0.143**	0.045*	*Dilution benefits; resource competition costs*
Body size, focal sp. × body size, target sp.	**0.492**	0.005**	*Dilution benefits; resource competition costs*
Group size, focal sp.	0.108	0.200	*Detection/dilution benefits; resource competition costs*
Group size, target sp.	0.062	0.343	*Detection/dilution benefits; resource competition costs*
Diet overlap	**−**0.133	0.065	*Resource competition costs*
Habitat overlap	0.012	0.856	*Control variable*
Similarity in movement pattern (resident/migratory)	**0.128**	0.021*	*Control variable*
Phylogenetic relatedness	**0.174**	0.011*	*Control variable*

Significant predictors (*P* < 0.05) are highlighted in bold (*P < 0.05; **P < 0.01).

a Social affinity indices modelled as a function of the traits of the focal and target species using multiple regression quadratic assignment procedure (MRQAP, 20 000 randomisations).

We calculated the social differentiation index according to Whitehead (2008) using a Poisson approximation to assess the degree of variation in affinities between species within the community.

All analyses were performed in R v. 3.4 (R Core Team 2016) with the packages *lme4* (Bates *et al.* 2015) and *asnipe* (Farine 2013) loaded.

## Results

**Figure 3:**
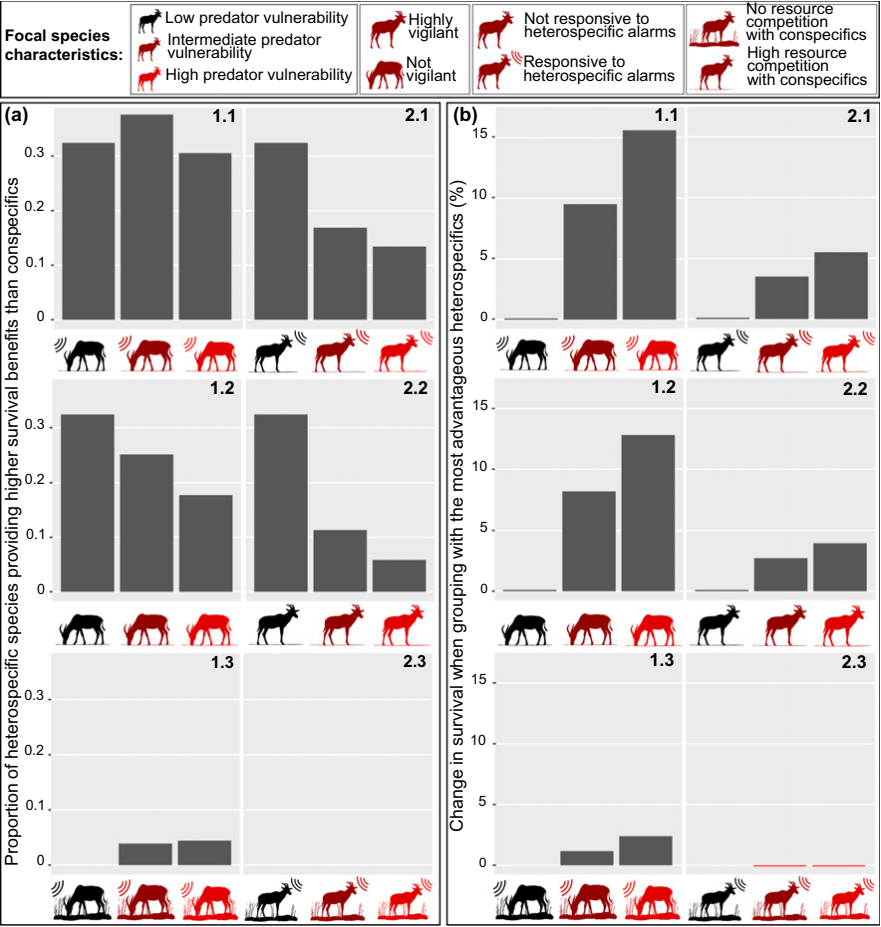
Theoretically predicted payoffs from joining heterospecifics rather than conspecifics. (a) The proportion of heterospecific species with whom grouping results in higher survival probability than obtained from conspecifics shown in relation to characteristics of the focal species. (b) Difference in survival probability between grouping with the most advantageous heterospecific species and grouping with conspecifics shown in relation to characteristics of the focal species. Predator vulnerability of the focal species increases from left to right in each diagram (as indicated by increasingly red colour). Diagrams 1 show results for species that are not vigilant (as indicated by lowered head), while diagrams 2 show results for vigilant species. Species in 1.1, 1.3, 2.1 and 2.3 respond to heterospecific alarm calls (as indicated by sound waves), whereas in 1.2 and 2.2 they do not. Species in 1.1, 1.2, 2.1 and 2.2 compete for resources with their conspecifics (as indicated by lack of grass), whereas in 1.3 and 2.3 they do not. Focal individuals were modelled in groups of various sizes (2, 5, 10, 20 and 40), which consisted either of conspecifics or one of a range of heterospecifics with different trait values.

**Figure 4:**
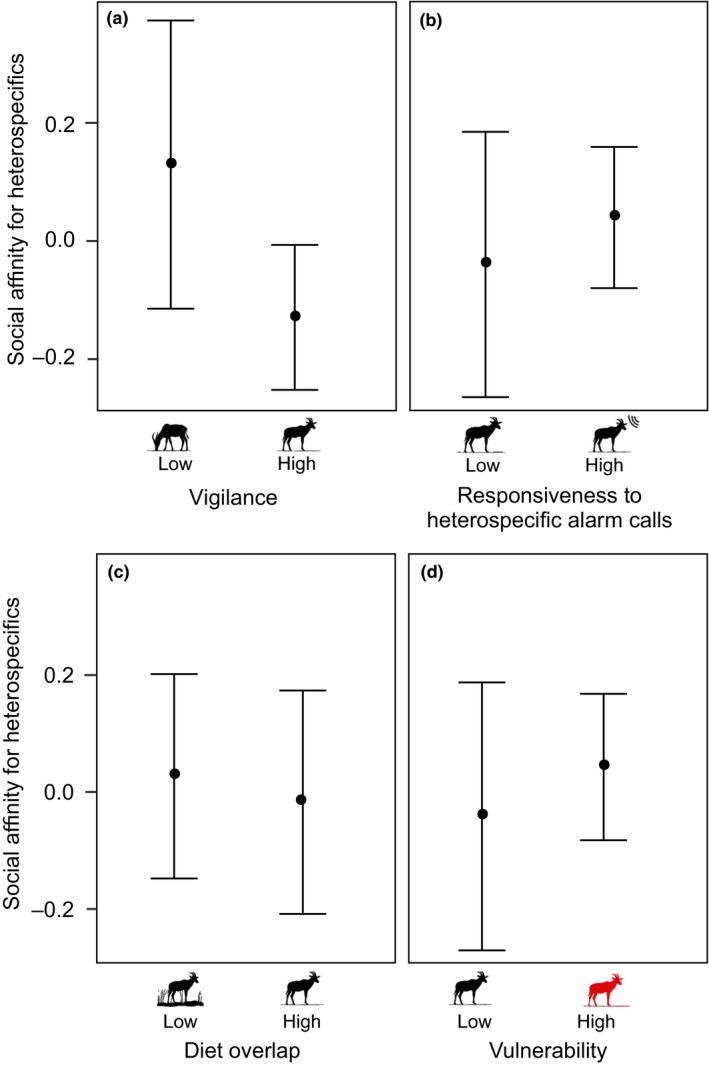
Social affinity for heterospecifics amongst African savannah herbivores in relation to species traits. (a) Vigilance, (b) responsiveness to heterospecific alarm calls, (c) diet overlap, and (d) predator vulnerability (using low body mass as a proxy for high predator vulnerability; Owen‐Smith & Mills 2008; Hopcraft et al. 2010). Graphs show the mean residual social affinity for the six species with the lowest, respectively highest, values for each trait (full analysis in Table 1). Error bars indicate the standard error of the mean.

**Figure 5:**
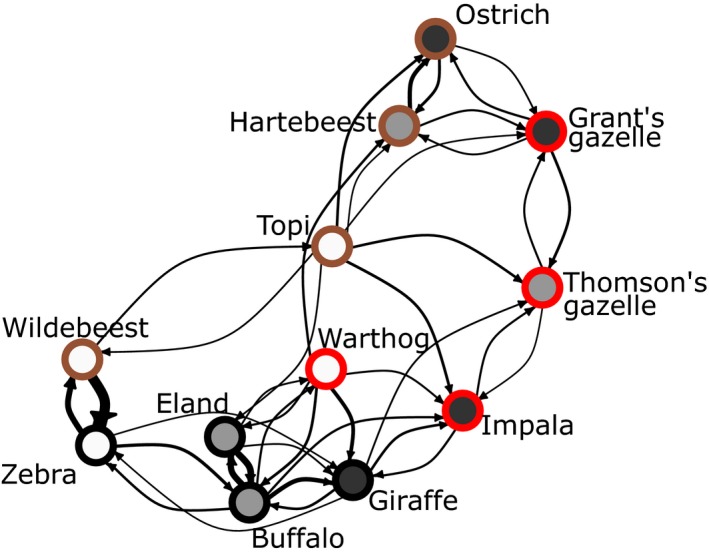
Weighted directed affinity network for the study species. Edge thickness indicates the strength of the affinity (only connections with weights over 0.1 shown). Node fill colour indicates vigilance (dark grey: high; light grey: intermediate; white: low). Node outline colour indicates overall predator vulnerability (red: high; brown: intermediate; black: low). The network illustrates differences between species in their affinity for heterospecifics as social partners; for instance, the attraction of buffaloes to the more vigilant giraffes is stronger than *vice versa*, and the attraction of warthogs to the more vigilant, but less vulnerable hartebeests is stronger than *vice versa*.

The ABM shows that decreasing vigilance of the focal species is associated with an increase in the proportion of heterospecific species with whom grouping results in a higher survival probability than obtained with conspecifics (Fig. 3a; compare diagrams 1 [not vigilant] to 2 [vigilant]). Also, the benefit obtained from grouping with the most advantageous of the heterospecific species is higher for focal species with low vigilance (Fig. 3b; compare diagrams 1 [not vigilant] to 2 [vigilant]). Our empirical study supports this theoretically predicted key role of vigilance for the formation of MSGs: among African savannah herbivores, social affinity for heterospecifics increased in species with relatively low vigilance (Table 1; Figs 4a & 5). An interaction term moreover shows that when more vigilant herbivores did form MSGs, they tended to specifically group with other vigilant species (Table 1).

The ABM also predicts an increased affinity for the heterospecifics with the most informative alarm calls (Fig. 3b; compare diagrams 1.1 resp. 2.1 [information transfer] to 1.2 resp. 2.2 [no information transfer]). This is again supported by our empirical study: social affinity was higher towards heterospecifics with more salient alarm calls, as measured by responsiveness in playback experiments (Table 1; Fig. 4b). In addition, the herbivore species who were less likely to alarm call in predator simulation experiments, showed a tendency towards higher social affinity for heterospecifics (Table 1); by contrast, the heterospecifics’ probability of alarm calling was not found to have any detectable impact on social affinity between the herbivore species (Table 1). These findings suggest that it is the ability of the receiver to extract relevant information from the heterospecific call (as measured by the strength of the alarm call response), rather than the overall probability that the heterospecific alarm calls, that best explains patterns in mixed‐species formation in the African savannah herbivore community.

The ABM further predicts that intense intraspecific resource competition enhances the attraction to heterospecifics rather than conspecifics as social partners (Fig. 3; compare diagrams 1.1 resp. 2.1 [high resource competition] to 1.3 resp. 2.3 [low resource competition]). Consistent with this, the empirical analysis reveals a weak tendency towards lower social affinity between herbivore species with a high degree of overlap in their diet (Table 1; Fig. 4c).

The ABM moreover shows that the benefits gained from grouping with the most advantageous heterospecific species increases with increasing predator vulnerability of the focal species (Fig. 3b). This pattern is more pronounced where resource competition is intense (Fig. 3b, compare diagrams 1.1 resp. 2.1 [high resource competition] to 1.3 resp. 2.3 [low resource competition]), which is attributable to heterospecifics generally inflicting lower resource competition costs than conspecifics. The empirical analysis agrees with the higher attraction to heterospecifics predicted for more vulnerable species in that smaller savannah herbivores, who are vulnerable to a wider range of predators (Owen‐Smith & Mills 2008; Hopcraft *et al.* 2010), were more likely to form MSGs (Table 1; Fig. 4d). An interaction term also shows that when the larger herbivore species did form MSGs, they were more likely to do so with other large species (Table 1), possibly because they are vulnerable to the same subset of predators (Sinclair *et al.* 2003).

In contrast to the high survival benefits that more vulnerable species get from grouping with the most advantageous heterospecifics, the proportion of heterospecific species that are preferable to conspecifics as social partners generally decreases with increasing predator vulnerability of the focal species in the ABM (Fig. 3a; diagrams 1.2, 2.1 & 2.2). This pattern can be explained by fewer heterospecific species providing dilution benefits above those of conspecifics in more vulnerable species. Thus, as the vulnerability of the focal species increases, the number of heterospecific species preferred over conspecifics decreases while the benefit of grouping with the most advantageous heterospecific increases. As a result, vulnerable species should be more selective regarding the heterospecific species with whom they chose to associate: most heterospecifics will not provide higher benefits than their own species, but certain heterospecifics will provide relatively large benefits. The social differentiation predicted from such selectivity is consistent with the Mara savannah herbivore community classifying as socially well‐differentiated according to a social differentiation index of 0.881 found in this study, with indices < 0.3 indicating homogenous communities, 0.3‐0.5 moderately differentiated communities, 0.5–2 well‐differentiated communities, and > 2.0 extremely differentiated communities (Whitehead 2008). (Note: an exception to the pattern of fewer heterospecifics being advantageous social partners as predator vulnerability of the focal species increases, is that the proportion of advantageous heterospecifics peaks at intermediate predator vulnerability in less vigilant species responsive to heterospecific alarm calls, thus indicating a shift in the optimal trade‐off between detection and dilution benefits [Fig. 3a; diagram 1.1]).

## Discussion

In this study, we used multi‐layered social network analysis to test theoretical predictions from an ABM designed to reveal how trade‐offs between detection and dilution benefits and resource competition costs result in assortment of species into MSGs according to their species characteristics. In our empirical study of African savannah herbivores, we found that species characterised by low vigilance had higher propensity to form MSGs, and that MSGs were more often formed with species whose alarm calls elicited strong responses. This is in accordance with the theoretical prediction of higher MSG benefits to less vigilant species and from joining heterospecifics with more informative alarm calls. These findings highlight the importance of detection benefits in promoting MSG formation and that dilution benefits alone are not enough to explain patterns in social affinity. Our study thus demonstrates social information use as a driver of interspecific sociality, in line with the general prediction made by Gil *et al *(2017) using dynamic state modelling.

Antipredator benefits from MSG formation are furthermore indicated by higher affinity for heterospecifics among smaller, more vulnerable, species. Conceivably, this affinity could also be promoted by a higher cost from intraspecific resource competition in smaller herbivores. Smaller ruminants have a higher rumen turnover rate due to a higher mass‐specific energy requirement (Kleiber (1975), and this necessitates an easily digestible, high protein diet (Demment & van Soest 1985). Such highly nutritious plant parts are less abundant than the fibrous forage which can be digested by larger species, and according to classical theory, their more dispersed food constrains the group size of smaller ruminant species (Jarman 1974). Hence, although the antipredator strategy of open‐habitat ungulates, regardless of body size, generally relies on safety‐in‐numbers, the typical size of single‐species groups is generally lower in smaller ruminants (Jarman 1974; Brashares *et al* 2000). As a consequence, smaller species may therefore experience higher payoffs from joining heterospecifics as they thereby can reduce overlap in resource use with other group members while still obtaining safety‐in‐numbers benefits.

In line with sensitivity to resource competition is also the tendency of the African savannah herbivores to avoid species with overlapping diet. Thus, although our results as a whole point to antipredator benefits as being central in shaping the overall patterns of interspecific social attraction among savannah herbivores, the impact of resource competition costs should not be dismissed. In this context, it should also be noted that we focussed on general patterns in social affinity averaged over one year, and it is likely that seasonal and interannual variation in food availability affects the role of resource competition costs relative to antipredator benefits, with consequences for the compositions of MSGs. However, whereas evidence from the study system indeed shows that patterns in social affinity undergo seasonal changes (Kiffner *et al.* 2014; Meise *et al.* 2019), the ABM‐predicted increase in heterospecific affinity when resources are scarce during the dry season was not evident from our seasonal analysis (Meise *et al.* 2019). The context‐dependent dynamics generated by seasonal variation in costs and benefits thus warrant further study.

By documenting the role of social information use in driving interspecific social associations, this study underscores that understanding social behaviour, not only within but also between species, can be of key importance in population and community ecology (Goodale *et al.* 2010; Farine *et al.* 2015b; Gil *et al.* 2017; Bro‐Jørgensen *et al.* 2019b). Information transfer between species has been shown to provide significant benefits in a wide range of other taxa, ranging from predator detection in vertebrates (Hetrick & Sieving 2012) to resource localisation in both vertebrates (Farine *et al.* 2015a) and invertebrates (Dawson & Chittka 2012), and the present findings bring attention to interspecific communication as a plausible driver of spatial organisation in these communities (Goodale *et al.* 2010; see also Martinez *et al.* 2018). However, social relations between species are often complex and likely to show a degree of system‐specificificity, and we therefore advocate community‐wide studies of contrasting systems to further test theory and gain a deeper understanding of the commonalities and differences in the drivers of interspecific sociality in different contexts. In need of further exploration are particularly the dynamics of MSG formation in systems where interactions over resources are believed to be mainly facilitative rather than competitive, such as in birds where ‘beating’ flushes insects to heterospecifics (Sridhar & Shanker 2014) or, as mentioned above, information is exchanged about the location of food resources. For this purpose, the ABM presented here can be easily adapted to reflect positive resource interactions. Although poorly quantified, facilitative processes also occur in ungulates where grazing modifies the vegetation layer to enhance resource availability for heterospecifics (McNaughton 1976; Bhola *et al.* 2012); however, a time‐lag before this facilitative effect manifest itself makes it less plausible as a main driver of MSG formation (see also Sinclair & Norton‐Griffiths 1982; Sinclair 1985). Still, when more accurate information becomes available on facilitative links, their incorporation into the ABM could potentially refine the fit between model predictions and empirical findings in our study as well. We note that our model, which includes the positive effect of shared vigilance on social affinity as a detection benefit, already implicitly accounts for what may be regarded as facilitative effects in cases where group vigilance, rather individual vigilance, is kept constant, thereby allowing feeding rate to be increased. The ABM can also be made more broadly applicable by allowing the formation of groups with more than two species, and modelling spatially explicit landscape‐scale networks may prove a useful approach in some systems (Hackett *et al.* 2019).

The significant impact that species can have on the survival of each other through their social links has clear implications for conservation management (Snijders *et al.* 2017), pointing to the limitations of single‐species approaches to predict population performance (Tylianakis *et al.* 2010; Valiente‐Banuet *et al.* 2015). Species‐focussed population viability analysis has long been the flagship approach to forecast population trends in conservation (Lacy 2019), but recently a call has been made for wider use of ecological function analysis which explicitly accounts for species roles within the ecosystem (Brodie *et al.* 2018). System‐wide repercussions consequential to population crashes of particular species may not always be obvious (Mokross *et al.* 2014; Gil & Hein 2017; Marthy & Farine 2018; Zou *et al.* 2018), and model frameworks incorporating social interactions between species, as the one presented here, can flag up cases of concern due to indirect effects of human‐induced perturbations; in particular the detrimental impact that loss of key informants can have, is evident from the present study. To more accurately predict socially‐mediated population responses, we now also need to know more about the plasticity of many social behaviours, which have evolved in relatively stable communities over evolutionary time: this plasticity will influence to what extent species can cope with the current rapid changes in many natural environments (Montiglio *et al.* 2018).

## Authorship

JBJ conceived and designed the study with inputs from DWF and KM. Field data were collected mainly by KM and secondarily by JBJ. KM performed the statistical analyses. The theoretical model was developed by DWF with significant input from KM and JBJ. JBJ wrote the manuscript based on a first draft written by KM and DWF.

## Data Availability

As stated in the Methods, the data are available from the NERC Environmental Information Data Centre (EIDC; Bro‐Jørgensen *et al.* 2019a; https://doi.org/10.5285/cc0794f0-748a-42aa-b491-2f9b65c771a6) and the Dryad Digital Repository: https://doi.org/10.5061/dryad.mb7dd20.
