# Supplementary material for: Alarm communication networks as a driver of community structure in African savannah herbivores
Source: Ecol Lett. 2019 Nov 27;23(2):293–304. doi: 10.1111/ele.13432 (PMC6973068; doi:10.1111/ele.13432)
Supplement: Supplementary file 1 [file ELE-23-293-s001.docx]

**SUPPORTING INFORMATION**

***Meise K, Franks DW & Bro-Jørgensen J:*** ***Alarm communication networks as a driver of community structure in African savannah herbivores***

**Table S1: Reference list for symbols in the agent-based model**

| *A_P_* | Probability of alarm calling in response to predator *P* |
| --- | --- |
| *C* | Individual resource consumption |
| *C_tot_* | Total amount of resources available |
| *c_1_, c_2,_ c_3_* | Constants |
| *D* | Probability of death due to predation |
| *E* | Phylogenetic relatedness |
| *F* | Focal species |
| *g* | Total number of groups |
| *H* | Habitat overlap between prey species |
| *i* | Identity of group |
| *J* | Overlap in movement pattern (migratory/resident) |
| *K* | Proportion of resources consumed by group (resource competition costs) |
| *L_P_* | Predator-specific probability of attacking a prey group upon encounter |
| *M* | Body mass |
| *N* | Group size |
| *O* | Diet overlap between prey species |
| *P* | Predator species |
| *p* | Total number of predator species |
| *Q_P_* | Predator-specific predation pressure (proportion of total prey biomass consumed) |
| *R* | Relevance of alarm call to receiver |
| *r* | Proportion of resource (browse/graze) in diet |
| *S* | Survival probability |
| *T* | Target species |
| *U_r_* | Proportion of resource *r* in the diet of a prey species |
| *V* | Vigilance |
| *W* | Social affinity |
| *X_P_* | Probability of detecting predator *P* (detection benefits) |
| *Y_P_* | Probability of not falling victim to an attack by predator *P* (dilution benefits) |
| *Z_P_* | Vulnerability of prey to predator *P* |

**Table S2: Parameters used in the agent-based model**

| **Species characteristic** | **Theoretical study** | **Empirical study^a^** |
| --- | --- | --- |
| Vigilance (*V*) | 0, 0.25, 0.50^b^ | 0.05 - 0.22 |
| Alarm call probability (*A*) | 0, 0.25, 0.50, 0.75, 1 | 0 - 1 |
| Responsiveness to alarm calls (*R*) | 0, 0.25, 0.50, 0.75, 1 | 0 - 1 |
| Predator vulnerability (*Z*) | 0, 0.25, 0.50, 0.75, 1 | 0 - 0.68 |
| Relative predation pressure (*Q)* | 1 | 0.02 - 0.64 |
| Resource consumption (*K*) | 0, 0.25, 0.50, 0.75, 1 | 0 - 1 |
| Group size (*N*) | 2, 5, 10, 20, 40 | 1 - 200 |

^a^See Table S3 for details.

^b^Vigilance above 0.50 was not modelled because the high tradeoff with resource acquisition in this case was considered to make it biologically less relevant (Beauchamp & Ruxton 2016).

**Table S3: Species-specific characteristics of the study species**

| **Species** | **Vigilance (sec/ min)** | **Body size*^a^***  **(kg)** | **Mean group size** | **Probability to alarm call** | | | | | **Predator vulnerability*^b^*** | | | | | **NDVI*^c^*** | **Diet (%)*^d^*** | |
| --- | --- | --- | --- | --- | --- | --- | --- | --- | --- | --- | --- | --- | --- | --- | --- | --- |
|  |  |  |  | **Jackal** | **Leopard** | **Cheetah** | **Hyena** | **Lion** | **Jackal** | **Leopard** | **Cheetah** | **Hyena** | **Lion** |  | **Monocots** | **Dicots** |
| **Thomson** | 9.4 | 21 | 4.94 | 0.895 | 0.984 | 0.964 | 0.971 | 0.96 | 0.38 | 0.57 | 0.665 | 0.27 | 0.19 | 4704 | 75 | 25 |
| **Grant** | 21.7 | 55 | 2.22 | 0.967 | 0.958 | 0.99 | 0.852 | 0.984 | 0.38 | 0.51 | 0.695 | 0.405 | 0.22 | 4373 | 65 | 35 |
| **Impala** | 17.4 | 53 | 6.93 | 0.53 | 0.907 | 0.57 | 0.844 | 0.98 | 0.38 | 0.68 | 0.555 | 0.455 | 0.135 | 5127 | 45 | 55 |
| **Warthog** | 8.0 | 74 | 2.28 | 0 | 0.463 | 0 | 0 | 0.731 | 0.15 | 0.4 | 0.295 | 0.4 | 0.555 | 4717 | 88.3 | 11.7 |
| **Topi** | 6.7 | 119 | 3.28 | 0.949 | 0.994 | 1 | 0.991 | 1 | 0 | 0.225 | 0.175 | 0.505 | 0.505 | 4762 | 95 | 5 |
| **Wildebeest** | 5.3 | 182 | 34.79 | 0.785 | 1 | 1 | 0.981 | 1 | 0.04 | 0.115 | 0.185 | 0.51 | 0.635 | 3359 | 87.5 | 12.5 |
| **Hartebeest** | 11.9 | 134 | 3.59 | 0.883 | 1 | 0.942 | 0.974 | 1 | 0.11 | 0.175 | 0.41 | 0.32 | 0.51 | 4328 | 75 | 25 |
| **Zebra** | 5.3 | 235 | 10.58 | 0 | 0.978 | 0.613 | 0.965 | 0.971 | 0.06 | 0.1 | 0.155 | 0.28 | 0.58 | 3135 | 92.6 | 7.4 |
| **Ostrich** | 17.5 | 121 | 3.66 | 0 | 0 | 0.677 | 0 | 0 | 0 | 0.18 | 0.195 | 0.295 | 0.225 | 5053 | 18.8 | 81.2 |
| **Giraffe** | 16.8 | 900 | 2.99 | 0.605 | 0.99 | 0.908 | 0.969 | 0.987 | 0 | 0.025 | 0.025 | 0.205 | 0.62 | 4811 | 0.8 | 99.2 |
| **Buffalo** | 8.6 | 631 | 11.39 | 0 | 0.709 | 0.728 | 0.641 | 0.913 | 0 | 0.08 | 0.03 | 0.305 | 0.66 | 5312 | 77.5 | 22.5 |
| **Eland** | 13.7 | 471 | 2.44 | 0 | 0 | 0 | 0 | 0 | 0.09 | 0.16 | 0.08 | 0.33 | 0.59 | 3745 | 50 | 50 |

^a^ Estes 2011, Deeming et al 1996

^b^ Hayward & Kerley 2005, Hayward 2006, Hayward et al. 2006a, 2006b, 2017

^c^ Note that the species-specific NDVI values are affected by seasonal difference in abundance, particularly for the migratory wildebeest and zebra.

^d^ Bro-Jørgensen 2013

**Table S4: Responsiveness to heterospecific alarm calls (PC1)**

| **Caller**  **Receiver** | **Thomson** | **Grant** | **Impala** | **Warthog** | **Topi** | **Wildebeest** | **Hartebeest** | **Zebra** | **Ostrich** | **Giraffe** | **Buffalo** | **Eland** |
| --- | --- | --- | --- | --- | --- | --- | --- | --- | --- | --- | --- | --- |
| **Thomson** | 0.95 | 0.93 | 0.85 | 0.55 | 0.93 | 0.90 | 0.59 | 0.90 | 0.71 | 0.47 | 0.60 | 0.00 |
| **Grant** | 0.79 | 0.95 | 0.68 | 0.65 | 0.85 | 0.76 | 0.58 | 0.84 | 0.58 | 0.54 | 0.51 | 0.00 |
| **Impala** | 0.72 | 0.87 | 0.95 | 0.74 | 0.76 | 0.84 | 0.96 | 0.99 | 0.53 | 0.50 | 0.65 | 0.00 |
| **Warthog** | 0.80 | 0.91 | 0.80 | 0.87 | 0.66 | 0.91 | 0.61 | 0.87 | 0.80 | 0.66 | 0.66 | 0.00 |
| **Topi** | 0.76 | 0.71 | 0.84 | 0.50 | 0.84 | 0.44 | 0.89 | 0.94 | 0.54 | 0.44 | 0.63 | 0.00 |
| **Wildebeest** | 0.73 | 0.82 | 0.50 | 0.26 | 0.71 | 0.76 | 0.56 | 0.74 | 0.26 | 0.04 | 0.01 | 0.00 |
| **Hartebeest** | 0.75 | 0.72 | 0.85 | 0.28 | 0.87 | 0.68 | 0.65 | 0.97 | 0.57 | 0.01 | 0.30 | 0.00 |
| **Zebra** | 0.77 | 0.84 | 0.87 | 0.67 | 0.59 | 0.91 | 0.57 | 0.87 | 0.39 | 0.17 | 0.50 | 0.00 |
| **Ostrich** | 0.65 | 0.87 | 0.86 | 0.78 | 0.42 | 0.71 | 0.69 | 0.94 | 0.93 | 0.92 | 0.82 | 0.00 |
| **Giraffe** | 0.65 | 0.65 | 0.98 | 0.43 | 0.69 | 0.56 | 0.60 | 1.00 | 0.24 | 0.60 | 0.63 | 0.00 |
| **Buffalo** | 0.09 | 0.51 | 0.29 | 0.21 | 0.50 | 0.24 | 0.19 | 0.36 | 0.15 | 0.27 | 0.38 | 0.00 |
| **Eland** | 0.78 | 0.76 | 0.56 | 0.46 | 0.78 | 0.39 | 0.29 | 0.63 | 0.43 | 0.33 | 0.30 | 0.00 |

**Table S5: Phylogenetic relationship (million years since last common ancestor)^a^**

|  | **Thomson** | **Grant** | **Impala** | **Warthog** | **Topi** | **Wildebeest** | **Hartebeest** | **Zebra** | **Ostrich** | **Giraffe** | **Buffalo** | **Eland** |
| --- | --- | --- | --- | --- | --- | --- | --- | --- | --- | --- | --- | --- |
| **Thomson** | NA | 10.6 | 23.2 | 65 | 23.2 | 23.2 | 23.2 | 80 | 300 | 33.2 | 25.4 | 25.4 |
| **Grant** | 10.6 | NA | 23.2 | 65 | 23.2 | 23.2 | 23.2 | 80 | 300 | 33.2 | 25.4 | 25.4 |
| **Impala** | 23.2 | 23.2 | NA | 65 | 22.3 | 22.3 | 22.3 | 80 | 300 | 33.2 | 25.4 | 25.4 |
| **Warthog** | 65 | 65 | 65 | NA | 65 | 65 | 65 | 80 | 300 | 65 | 65 | 65 |
| **Topi** | 23.2 | 23.2 | 22.3 | 65 | NA | 10.8 | 10.8 | 80 | 300 | 33.2 | 25.4 | 25.4 |
| **Wildebeest** | 23.2 | 23.2 | 22.3 | 65 | 10.8 | NA | 10.8 | 80 | 300 | 33.2 | 25.4 | 25.4 |
| **Hartebeest** | 23.2 | 23.2 | 22.3 | 65 | 10.8 | 10.8 | NA | 80 | 300 | 33.2 | 25.4 | 25.4 |
| **Zebra** | 80 | 80 | 80 | 80 | 80 | 80 | 80 | NA | 300 | 80 | 80 | 80 |
| **Ostrich** | 300 | 300 | 300 | 300 | 300 | 300 | 300 | 300 | NA | 300 | 300 | 300 |
| **Giraffe** | 33.2 | 33.2 | 33.2 | 65 | 33.2 | 33.2 | 33.2 | 80 | 300 | NA | 33.2 | 33.2 |
| **Buffalo** | 25.4 | 25.4 | 25.4 | 65 | 25.4 | 25.4 | 25.4 | 80 | 300 | 33.2 | NA | 18.3 |
| **Eland** | 25.4 | 25.4 | 25.4 | 65 | 25.4 | 25.4 | 25.4 | 80 | 300 | 33.2 | 18.3 | NA |

^a^ Benton 1990; Fernández & Vrba, 2005; Gatesy et al. 2013

**Table S6: Undirected affinity indices**

|  | **Thomson** | **Grant** | **Impala** | **Warthog** | **Topi** | **Wildebeest** | **Hartebeest** | **Zebra** | **Ostrich** | **Giraffe** | **Buffalo** | **Eland** |
| --- | --- | --- | --- | --- | --- | --- | --- | --- | --- | --- | --- | --- |
| **Thomson** | NA | 2.067 | 1.557 | 0.830 | 1.383 | 0.718 | 0.788 | 0.542 | 0.761 | 1.405 | 0.556 | 0.762 |
| **Grant** | 2.067 | NA | 0.595 | 0.719 | 0.966 | 0.359 | 1.719 | 0.791 | 1.723 | 0.425 | 0.080 | 0.792 |
| **Impala** | 1.557 | 0.595 | NA | 1.288 | 1.700 | 0.549 | 1.017 | 0.608 | 0.149 | 2.037 | 0.746 | 1.505 |
| **Warthog** | 0.830 | 0.719 | 1.288 | NA | 1.031 | 0.550 | 1.639 | 0.362 | 0.180 | 2.218 | 1.669 | 2.091 |
| **Topi** | 1.383 | 0.966 | 1.700 | 1.031 | NA | 1.628 | 0.971 | 1.091 | 1.430 | 0.605 | 1.234 | 0.654 |
| **Wildebeest** | 0.718 | 0.359 | 0.549 | 0.550 | 1.628 | NA | 0 | 10.491 | 0.292 | 0 | 0.218 | 0.425 |
| **Hartebeest** | 0.788 | 1.719 | 1.017 | 1.639 | 0.971 | 0 | NA | 0.770 | 3.031 | 0 | 0 | 0 |
| **Zebra** | 0.542 | 0.791 | 0.608 | 0.362 | 1.091 | 10.491 | 0.770 | NA | 0.442 | 1.338 | 0.578 | 2.435 |
| **Ostrich** | 0.761 | 1.723 | 0.149 | 0.180 | 1.430 | 0.292 | 3.031 | 0.442 | NA | 0 | 0 | 0 |
| **Giraffe** | 1.405 | 0.425 | 2.037 | 2.218 | 0.605 | 0 | 0 | 1.338 | 0 | NA | 1.206 | 3.014 |
| **Buffalo** | 0.556 | 0.080 | 0.746 | 1.669 | 1.234 | 0.218 | 0 | 0.578 | 0 | 1.206 | NA | 5.539 |
| **Eland** | 0.762 | 0.792 | 1.505 | 2.091 | 0.654 | 0.425 | 0 | 2.435 | 0 | 3.014 | 5.539 | NA |

**Table S7: Directed affinity indices**

| **Target**  **Focal** | **Thomson** | **Grant** | **Impala** | **Warthog** | **Topi** | **Wildebeest** | **Hartebeest** | **Zebra** | **Ostrich** | **Giraffe** | **Buffalo** | **Eland** |
| --- | --- | --- | --- | --- | --- | --- | --- | --- | --- | --- | --- | --- |
| **Thomson** | NA | 0.129 | 0.096 | 0.030 | 0.042 | 0.013 | 0.044 | 0.000 | 0.053 | 0.093 | 0.019 | 0.025 |
| **Grant** | 0.171 | NA | 0.042 | 0.034 | 0.040 | 0.011 | 0.117 | 0.023 | 0.141 | 0.033 | 0.004 | 0.037 |
| **Impala** | 0.130 | 0.045 | NA | 0.065 | 0.075 | 0.024 | 0.073 | 0.032 | 0.012 | 0.138 | 0.043 | 0.077 |
| **Warthog** | 0.091 | 0.070 | 0.121 | NA | 0.068 | 0.033 | 0.151 | 0.026 | 0.019 | 0.231 | 0.130 | 0.168 |
| **Topi** | 0.159 | 0.100 | 0.172 | 0.081 | NA | 0.106 | 0.096 | 0.092 | 0.164 | 0.065 | 0.107 | 0.057 |
| **Wildebeest** | 0.091 | 0.041 | 0.055 | 0.047 | 0.130 | NA | 0 | 1.000 | 0.030 | 0 | 0.021 | 0.040 |
| **Hartebeest** | 0.070 | 0.132 | 0.075 | 0.087 | 0.045 | 0 | NA | 0.032 | 0.262 | 0 | 0 | 0 |
| **Zebra** | 0.079 | 0.092 | 0.056 | 0.027 | 0.066 | 0.522 | 0.080 | NA | 0.036 | 0.098 | 0.051 | 0.200 |
| **Ostrich** | 0.057 | 0.109 | 0.009 | 0.007 | 0.044 | 0.012 | 0.178 | 0.028 | NA | 0 | 0 | 0 |
| **Giraffe** | 0.111 | 0.029 | 0.158 | 0.091 | 0.022 | 0 | 0 | 0.096 | 0 | NA | 0.067 | 0.159 |
| **Buffalo** | 0.061 | 0.008 | 0.066 | 0.112 | 0.072 | 0.011 | 0 | 0.032 | 0 | 0.108 | NA | 0.414 |
| **Eland** | 0.085 | 0.078 | 0.141 | 0.135 | 0.037 | 0.022 | 0 | 0.153 | 0 | 0.278 | 0.390 | NA |

**Table S8: Species characteristics as predictors of social affinity between species in an undirected affinity network (cfr. Table 1). Significant predictors (*P*<0.05) are highlighted in bold (* *P*<0.05; ** *P*<0.01).**

| **Trait** | **Coefficient** | ***P*-value** | **Related costs/benefits** |
| --- | --- | --- | --- |
| Vigilance rate^a^ | **-0.713** | **0.030*** | *Detection benefits* |
| Responsiveness to alarm call^a^ | **1.267** | **0.006**** | *Detection benefits* |
| Alarm call probability^a^ | -0.719 | 0.118 | *Detection benefits* |
| Body size^a^ | 0.835 | 0.053 | *Dilution benefits; resource competition costs* |
| Body size difference | **-0.642** | **0.015*** | *Dilution benefits; resource competition costs* |
| Group size^a^ | 0.935 | 0.063 | *Detection/dilution benefits; resource competition costs* |
| Diet overlap | -0.217 | 0.483 | *Resource competition costs* |
| Habitat overlap | -0.105 | 0.758 | *Control variable* |
| Movement pattern similarity | **0.763** | **0.011*** | *Control variable* |
| Phylogenetic relatedness | -0.562 | 0.077 | *Control variable* |

^a^Mean of the focal and target species’ trait values.

**References**

Beauchamp G., and Ruxton G.D. (2016). Modeling scan and interscan durations in antipredator vigilance. *J. Theor. Biol.* 390, 86-96.

Benton M.J. (1990). Phylogeny of the major tetrapod groups: morphological data and divergence dates. *J. Mol. Evol.* 30, 409-424.

Bro-Jørgensen, J. (2013). Evolution of sprint speed in African savannah herbivores in relation to predation. *Evolution* 67, 3371–3376.

Estes R.D. (2011). *The Behavior Guide to African Mammals.* 2^nd^ edn. University of California Press, Berkeley.

Deeming D.C., Sibly, R.M., and Magole, I.L. (1996). Estimation of the weight and body condition of ostriches (*Struthio camelus*) from body measurements. *Vet. Rec.* 139, 210–213.

Fernández M.H., and Vrba E.S. (2005). A complete estimate of the phylogenetic relationships in Ruminantia: a dated species-level supertree of the extant ruminants. *Biol. Rev.* 80, 269-302.

Gatesy J., Geisler J.H., Chang J., Buell C., Berta A., Meredith R.W., Springer M.S., and McGowen M.R. (2013). A phylogenetic blueprint for a modern whale. *Mol. Phylogenet. Evol.* 66, 479-506.

Hayward M.W. (2006). Prey preferences of the spotted hyaena (*Crocuta crocuta*) and degree of dietary overlap with the lion (*Panthera leo*). *J. Zool.* 270, 606-614.

Hayward M.W., and Kerley G.I.H. (2005). Prey preferences of the lion (*Panthera leo*). *J. Zool.* 267, 309.

Hayward M.W., Henschel P., O’Brien J., Hofmeyr M., Balme G., and Kerley G.I.H. (2006a). Prey preferences of the leopard (*Panthera pardus*). *J. Zool.* 270, 298-313.

Hayward M.W., Hofmeyr M., O’Brien J., and Kerley G.I.H. (2006b). Prey preferences of the cheetah (*Acinonyx jubatus*) (Felidae: Carnivora): morphological limitations or the need to capture rapidly consumable prey before kleptoparasites arrive? *J. Zool.* 270, 615–627.

Hayward M.W., Porter L., Lanszki J., Kamler J.F., Beck J.M., Kerley G.I.H., Macdonald D.W., Montgomery R.A., Parker D.M., Scott D.M., O’Brien J., and Yarnell R.W. (2017). Factors affecting the prey preferences of jackals (Canidae). *Mamm. Biol.* 85, 70–82.
